# Sleep habits and orofacial myofunctional self-assessment of children at risk for sleep breathing disorders

**DOI:** 10.1590/2317-1782/20232022187en

**Published:** 2023-12-22

**Authors:** Melissa Picinato-Pirola, Amanda Lima e Lira, Giovanna Régis Viana, Thaynara Lemos Batista Santos, Camila de Castro Corrêa

**Affiliations:** 1 Faculdade de Ceilândia, Universidade de Brasília - UnB - Brasília (DF), Brasil.; 2 Centro Universitário Planalto do Distrito Federal - UNIPLAN - Brasília (DF), Brasil.

**Keywords:** Child, Stomatognathic System, Sleep Apnea, Obstructive, Sleep Disorders, Speech, Language and Hearing Sciences

## Abstract

**Purpose:**

To identify orofacial myofunctional complaints and sleep-disordered breathing and correlate them with sleep habits in childhood.

**Methods:**

The study included 71 parents/guardians of public school children aged 6 to 11 years. They answered a form with semi-structured medical history questions and the Nordic Orofacial Test-Screening - interview, the Pediatric Obstructive Sleep Apnea Screening Tool Questionnaire, and the Children’s Sleep Habits Questionnaire - all of them in their Portuguese/Brazilian versions in an online format. Statistical analyses used Spearman’s correlation, setting the significance level at 5%.

**Results:**

There were 29 female children (40.8%) and 42 male ones (59.2%), with a mean age of 8.52 years. The study found orofacial myofunctional complaints related to breathing functions (35.2%), chewing and swallowing (32.4%), and deleterious habits (33.8%). All children were at a low risk of sleep-disordered breathing. As for sleep habits, 23 children (32.39%) had a lower total score, whereas 48 children (67.61%) exceeded 41 points.

**Conclusion:**

There was a correlation between the risk of sleep-disordered breathing in children with complaints of orofacial myofunctional disorders and poor sleep quality/habits.

## INTRODUCTION

Sleep-disordered breathing (SDB) encompasses aspects characterized by upper airway resistance syndrome, snoring, and obstructive and central sleep apnea^(1)^. The latter is due to routine episodes of partial or total interruption of airflow in the upper respiratory tract caused by an anatomical obstruction in pharyngeal lymphoid tissue, making it collapse during sleep^(2)^. In children, its main causes include hypertrophy of pharyngeal and palatine tonsils, obesity/overweight, neuromotor diseases, and syndromes or changes related to the craniofacial complex^(1-4)^. These aspects can impair children’s global development, especially at school age when it is difficult to establish a sleep routine, compromising their weight, craniofacial, cardiopulmonary, neuropsychomotor, and behavioral growth, besides excessive daytime sleepiness and other disorders and habits unfavorable to restful sleep^(3,5,6)^.

Polysomnography (PSG) is the worldwide reference objective examination to diagnose SDB. However, its high cost and inaccessibility for a large portion of the world population have led science to increasingly advance in recent years in developing standardized questionnaires able to screen the risks of SDB and other sleep disorders. Such progress seems favorable when considering the priority of these examinations and the importance of early treatment when necessary^(7-11)^ - although screening questionnaires do not substitute diagnosis with PSG.

Adenotonsillectomy is known to be the gold standard treatment of pediatric obstructive SDB^(1)^. Nonetheless, studies show that even after surgery, many children continue having functional changes in their stomatognathic system due to their old preintervention orofacial patterns^(12)^, with changes in their craniofacial and muscle structure^(13)^, as well as in breathing, mastication, swallowing, and speech^(14,15)^.

Thus, structured orofacial myofunctional assessment and follow-up with orofacial myofunctional therapy are necessary, even after adenotonsillectomy, essentially for children to achieve a good quality of life^(12,15)^. Given the above issues, this study aimed to correlate complaints of orofacial myofunctional changes and risks of SDB with children’s sleep habits.

## METHODS

This quantitative, cross-sectional, observational study had a sample of 71 parents/guardians of public school children of both sexes, aged 6 to 11 years, attending from kindergarten to fifth grade between October 2020 and April 2021. The inclusion criteria were students properly enrolled in the participating school, aged 6 to 11 years, whose parents/guardians agreed with their participation by signing an informed consent form and filling out the entire form. The research was approved by the Human Research Ethics Committee of the University of Brasília - Campus of Ceilândia (UnB - FCE), under CAAE number 33422820.4.0000.8093 and evaluation report number 4.227.063. The study excluded children who were taking sleep-inducing drugs and who had been diagnosed with attention-deficit/hyperactivity disorder, pervasive developmental disorders, or any syndrome that might impair their craniofacial and/or neurocognitive development, as well as those whose parents/guardians did not volunteer, did not sign an informed consent form, or filled out the questionnaires incompletely.

Data were collected remotely in a single stage through a form administered via Google Forms. The research was adapted to online data collection due to the social isolation safety measures to cope with the COVID-19 pandemic. Thus, the participating school’s coordinator was initially contacted via phone call to survey the students in the intended age range and send the form via e-mail to their parents/guardians. Hence, all parents/guardians of the children enrolled in that school were invited to fill out semi-structured questions essentially on age, sex, weight, height (to calculate the body mass index [BMI]), overall health, respiratory diseases, medications, nutritional aspects, syndromes, and mastication and swallowing difficulties. The same form also had the following three protocols, separated per category: the Nordic Orofacial Test-Screening (NOT-S) - interview, translated and validated in Brazilian Portuguese^(16)^; the Pediatric Obstructive Sleep Apnea Screening Tool Questionnaire (PosaST) - translated and validated in Brazilian Portuguese^(11)^; and the Children’s Sleep Habits Questionnaire (CSHQ-PT), Portuguese version^(5)^.

NOT-S can be applied to various age ranges to indicate complaints of orofacial myofunctional changes. It has the following two parts: initial interview, addressing aspects related to (I) sensory function, (II) breathing, (III) habits, (IV) mastication and swallowing, (V) salivation, and (VI) mouth dryness, as well as the clinical examination. This research used only the interview to screen possible oral myofunctional complaints. Each domain has one to five items that must be checked with an “X” (indicating a change is present) or “0” (indicating its absence), with scores ranging from 0 to 6 (1 to 12 in the complete protocol) - higher scores indicate concern^(16)^.

PosaST - Brazilian version is highly sensitive to screen complaints of obstructive SDB in schoolchildren. It is basically an interview/questionnaire administered to the parents/guardians of children with SDB symptoms. It has six questions whose response patterns are based on the frequency with which episodes occur: “never” (0), “rarely” (once a week), “occasionally” (twice a week), “often” (three to four times a week); and “almost always” (more than four times a week). The item on snoring intensity, however, is scored as follows: “low” (0), “somewhat high” (1), “high” (2), “very high” (3), and “extremely high” (4). The mean of the six questions is calculated with the formula, A = (Q1+Q2)/2; B = (A+Q3)/2; C = (B+Q4)/2; D = (C+Q5)/2. The final analysis considers the summed score = (D+Q6)/2. Q1 refers to question 1 and so forth; scores ≥ 2.72 are suggestive of a high risk of SDB^(11)^.

CSHQ-PT has been adapted to Portuguese and aims to assess sleep habits in children aged 2 to 10 years, according to the parents’ perception in the previous week or the one nearest to the assessment. This version has 33 items with eight subscales that reflect the following sleep domains: (1) resistance to go to bed (items 1, 3, 4, 5, 6, and 8); (2) sleep onset; (3) sleep duration (items 9, 10, and 11); (4) sleep anxiety (items 5, 7, 8, and 21); (5) nighttime wakefulness (items 16, 24, and 25); (6) parasomnia (items 12, 13, 14, 15, 17, 22, and 23); (7) SDB (items 18, 19, and 20); and (8) daytime sleepiness (items 26, 27, 28, 29, 30, 31, 32, and 33). The frequency with which these behaviors occur is classified as follows: habitually (5-7 times a week; total of 3 points); sometimes (2-4 times a week, total of 2 points), and rarely (0-1 time a week, total of 1 point). Items 1, 2, 3, 10, 11, and 26 have inverted scores - i.e., higher scores indicate greater sleep changes. The cutoff was set at 41 points to detect sleep disorders^(5)^. The analysis considered the mean and standard deviation of the sum of each subscale and the mean final score.

Statistical data analyses were described in means, standard deviations, and percentages. The Spearman correlation was applied to the final NOT-S, CSHQ-PT, and PosaST scores to correlate the findings and complaints of myofunctional changes, sleep, and SDB. The software used was SPSS, version 23, and the significance level was set at 5%.

## RESULTS

According to statistics, the study included 71 parents/guardians of schoolchildren - 29 females (40.8%) and 42 males (59.2%), with a mean age of 8.52 years. The mean BMI was 17.73±4.39 kg/m^2^.

The medical history survey verified health problems, among which the most reported were night mouth breathing (15.5%), snoring (11.2%), asthma (9.9%), and sinusitis (8.5%).

The main orofacial myofunctional complaints found with NOT-S were in the domains of breathing (35.2%), habits (33.8%), and mastication and swallowing (32.4%). The results are shown in Table 1.

**Table 1:** Distribution of the sample regarding orofacial myofunctional complaints reported by the parents/guardians in the Nordic Orofacial Test- Screening (NOT-S)

**NOT-S**	**Presence of complaints**
**Domains**	
Sensory function	13 (18.3%)
Breathing	25 (35.2%)
Habits (deleterious)	24 (33.8%)
Mastication and swallowing	23 (32.4%)
Salivation	1 (1.4%)
Mouth dryness	10 (14.1%)
**Total domains**	**Mean±SD**
	1.35±1.23

**Caption:** NOT-S = Nordic Orofacial Test-Screening; SD = standard deviation.

PosaST found that all the children were at low risk of SDB (Table 2). As for sleep habits verified with CSHQ-PT, the sample mean score was above the cutoff, characterizing the possibility of sleep disorders (Table 3). Specifically, 23 children (32.39%) had a lower sum, whereas 48 children (67.61%) were above 41 points.

**Table 2:** Analysis of the Pediatric Obstructive Sleep Apnea Screening Tool (PosaST) questions concerning the risk of children’s sleep breathing disorders

**PosaST questions**	**Mean±SD**	**Minimum**	**Maximum**
**Q1**	0.13±0.41	0	2
**Q2**	0.27±0.53	0	2
**Q3**	0.03±0.17	0	1
**Q4**	0.77±1.14	0	4
**Q5**	0.44±0.79	0	3
**Q6**	0.20±0.43	0	2
**Formulas indicated by the questionnaire**			
**A**	0.14±0.40	0	2.00
**B**	0.04±0.19	0	1.25
**C**	0.32±0.69	0	2.62
**D**	0.38±0.67	0	1.75
**Total score**	0.27±0.53	0	1.38

**Caption**: SD = standard deviation; Q1 = Does your child stop breathing during sleep?; Q2 = Does your child have difficulties breathing during sleep?; Q3 = Have you ever had to shake your child to make them start breathing again during sleep?; Q4 = How often does your child snore?; Q5 = Do you have any concern about your child’s breathing while they sleep?; Q6 = How high is your child’s snoring?; A = (Q1+Q2)/2; B = (A+Q3)/2; C = (B+Q4)/2; D = (C+Q5)/2; total score = (D+Q6)/2.

**Table 3:** Distribution of the sample regarding sleep-related habits according to the Children’s Sleep Habits Questionnaire (CSHQ-PT)

**CSHQ-PT subscales**	**Mean±SD**	**Minimum**	**Maximum**
**Resistance to go to bed**	8.97±3.24	6	17
**Sleep onset**	1.97±0.89	1	3
**Sleep duration**	3.83±1.37	3	9
**Anxious sleep**	6.07±1.89	4	12
**Nighttime wakefulness**	3.83±1.18	3	9
**Parasomnia**	8.94±1.80	7	13
**Sleep breathing disorders**	3.62±1.06	3	7
**Daytime sleepiness**	10.82±2.42	8	16
**Total score**	44.90±8.20	33	66

Subscale (1) resistance to go to bed (sum of items 1, 3, 4, 5, 6, and 8); Subscale (2) sleep onset; Subscale (3) sleep duration (items 9, 10, and 11); Subscale (4) anxious sleep (items 5, 7, 8, and 21); Subscale (5) nighttime wakefulness (items 16, 24, and 25); subscale (6) parasomnia (items 12, 13, 14, 15, 17, 22, and 23); Subscale (7) sleep breathing disorders (items 18, 19, and 20); and Subscale (8) daytime sleepiness (sum of items 26, 27, 28, 29, 30, 31, 32, and 33).

**Caption:** SD = standard deviation.

The inferential analysis verified a positive relationship between the risk of developing obstructive SDB (PosaST) and orofacial myofunctional complaints (NOT-S) (p = 0.033). Moreover, the risk of obstructive SDB (PosaST) was related to sleep changes (CSHQ-PT) (p = 0.007), which in turn was positively related to orofacial myofunctional complaints (NOT-S) (p = 0.000). The results are shown in Table 4.

**Table 4:** Correlation between scores of the Nordic Orofacial Test- Screening (NOT-S), Pediatric Obstructive Sleep Apnea Screening Tool (PosaST), and Children’s Sleep Habits Questionnaire (CSHQ-PT)

	**Mean±SD**	**PosaST**	**NOT-S**	**CSHQ-PT**
**1.PosaST**	0.197±0.434		0.254	0.319
			0.033*	0.007*
**2.NOT-S**	1.535±1.519			0.438
				0.000*
**3.CSHQ-PT**	1.360±0.248			

Statistical test: Spearman Correlatio

* Significance = p < 0.05

**Caption:** PosaST = Pediatric Obstructive Sleep Apnea Screening Tool; NOT-S = Nordic Orofacial Test Screening; CSHQ-PT = Children Sleep Habits Questionnaire - Portuguese version; SD = standard deviation; the first datum is R = proportionality coefficient of the correlation; the second line has Spearman correlation’s p, considering p < 0.05.

## DISCUSSION

This study aimed to survey orofacial myofunctional complaints related to SDB in schoolchildren, demonstrating their correlation with SDB complaints and sleep habits. Therefore, speech-language-hearing therapists must work interdisciplinarily with related sleep specialization areas to diagnose and follow up on children with sleep complaints, which can coexist with orofacial function complaints.

According to the literature, school and preschool children at risk of SDB usually snore^(3,17-19)^. Even though the sample in this study was at a low risk of SDB, the results were similar to those in the literature.

Concerning the main orofacial myofunctional complaints found in Table 1, a study used NOT-S in Brazilian public school children aged 8 to 10 years but did not find a direct association with SDB. The interview verified that 50.8% of the children had difficulties masticating solid foods, and 24.4% of them snored^(20)^. The study also showed that mastication and swallowing (50.5%), habits (41.4%), and breathing (26.4%)^(21)^ were the most compromised domains, demonstrating that these aspects can impair child development. Despite the lack of a direct association with SDB, these findings were similar to the ones in the present study.

Another previous study assessed children aged 6 to 11 years diagnosed with obstructive sleep apnea (OSA) and primary snoring, using the Orofacial Myofunctional Evaluation Protocol with Scores (OMES), PSG, and electromyography. It verified that children with OSA had changes in masticatory muscle strength/contraction, posture, mobility, and coordination, compromising their good performance in mastication, swallowing, and breathing (mostly mouth breathing)^(12)^. The present study did not use objective examinations and assessment protocols to determine the aspect of abnormal orofacial functions, and its participants were not divided into experimental and control groups.

PosaST Brazilian version, administered in the present study, verified that all its children were at low risk of SDB, as described in the Brazilian validation study^(11)^. PosaST proved to be rather sensitive to screening particularly mild SDB symptoms, which tend to improve in 80% of the cases with medications and/or orofacial myofunctional therapy, without the need for adenotonsillectomy. This instrument was included in the present study for its high sensitivity and specificity and for being an easily applied screening instrument. So far, no other study was found in Brazil applying PosaST (except for the Brazilian validation one), and only one SDB screening questionnaire was found - although it included not only school and preschool children but also adolescents^(22)^, who were not included in the present study.

Questionnaires on child sleep habits and disorders have been developed as a screening alternative to PSG and especially to complement the findings of medical history surveys and clinical assessments^(5,8,11)^. The present study used the term SDB because, according to international guidelines and systematic reviews, it is the one used to refer to a group of respiratory manifestations that occur during sleep, interrupting the airflow in the upper airways, and causing micro-wakes^(1,9)^. The present research specifically used CSHQ-PT, which identified a lower occurrence of signs indicative of SDB than in the literature^(3)^.

All protocols used in the present study were mutually correlated. However, no studies were found in the literature applying together at least two of these three protocols - in which sense this study is innovative. Thus, the greater the risk of SDB, the greater the impairments in orofacial functions. A study in 86 Brazilian and Italian children aged 4 to 11 years used instruments to screen SDB and orofacial complaints (though not PosaST or NOT-S). It verified that children with SDB had changes in the craniofacial complex and consequently in orofacial functions, demonstrating the existing relationship between these two aspects^(23)^. Another study likewise demonstrated that SDB complaints are associated with orofacial myofunctional changes^(24)^, as highlighted in the present study.

The aspects approached in the relationship between PosaST and CSHQ-PT demonstrated that the higher the risk of SDB, the greater the impairment in the quality of sleep. This is due to impaired sleep latency, duration, and efficiency patterns in children with SDB, causing excessive daytime sleepiness and multiple wakes, as reported in the literature^(1,6)^.

The correlation between NOT-S and CSHQ-PT scores demonstrated that sleep disturbances directly influence the balance of orofacial functions. The literature has similar findings, such as in a Brazilian study that used NOT-S to investigate orofacial myofunctional complaints. Using the Pittsburgh Sleep Quality Index (instead of CSHQ-PT), it found that 38% of the sample had a low sleep quality^(25)^. Hence, all aspects reported above are closely interconnected and help negatively impact schoolchildren’s quality of life and development^(5,26)^.

The limitations of this study are mainly related to not dividing the children into control and experimental groups, as all of them were at a low risk of SDB. Another factor with a great impact was the impossibility of collecting data in person (due to the measures taken to cope with the COVID-19 pandemic), preventing the use of the clinical assessment in NOT-S to determine more precisely the nature of the orofacial changes.

Future studies should use objective assessments and examinations to determine the diagnosis of orofacial dysfunctions in larger samples. They should also divide the sample into experimental and control groups to better determine the prevalence of the risk of SDB and its implications for the quality of children’s sleep and characteristics related to the stomatognathic system.

## CONCLUSION

The risk of SDB in children was correlated with complaints of orofacial myofunctional changes and poor sleep quality/habits. This study's main orofacial myofunctional complaints were related to the respiratory system. These complaints may be associated with a possible risk of SDB, compromising the balance of orofacial functions and sleep quality in schoolchildren.
