# Supplementary material for: Sleep habits and orofacial myofunctional self-assessment of children at risk for sleep breathing disorders
Source: Codas. 2023 Dec 22;36(1):e20220187. doi: 10.1590/2317-1782/20232022187en (PMC10750857; doi:10.1590/2317-1782/20232022187en)
Supplement: Translation

# Translation: Hábitos de sono e autoavaliação miofuncional orofacial de crianças com risco para distúrbios respiratórios do sono

**DOI:** 10.1590/2317-1782/20232022187pt

**Authors:** Melissa Picinato-Pirola, Amanda Lima e Lira, Giovanna Régis Viana, Thaynara Lemos Batista Santos, Camila de Castro Corrêa

**Affiliations:** 1 Faculdade de Ceilândia, Universidade de Brasília - UnB - Brasília (DF), Brasil.; 2 Centro Universitário Planalto do Distrito Federal - UNIPLAN - Brasília (DF), Brasil.

**Keywords:** Criança, Sistema Estomatognático, Apneia Obstrutiva do Sono, Transtornos do Sono, Fonoaudiologia

## Abstract

**Objetivo:**

Identificar e correlacionar as queixas de alterações miofuncionais orofaciais e de risco para distúrbios respiratórios do sono com hábitos de sono na infância.

**Método:**

Participaram 71 pais ou responsáveis de crianças de 6 a 11 anos, matriculadas em uma escola pública. Foi aplicado um formulário com questões semiestruturadas/anamnese e os protocolos Nordic Orofacial Test-Screening - entrevista, Pediatric Obstructive Sleep Apnea Screening Tool Questionnaire, e Children’s Sleep Habits Questionnaire - todos em suas versões em português/Brasil no formato online. Para a análise estatística, realizou-se o teste de correlação de Spearman, considerando o nível de significância de 5%.

**Resultados:**

29 crianças eram do sexo feminino (40,8%) e 42 do sexo masculino (59,2%), com média de idade de 8,52 anos. As queixas miofuncionais orofaciais encontradas foram relacionadas às funções de respiração (35,2%), mastigação e deglutição (32,4%) e hábitos deletérios (33,8%). Todas as crianças apresentaram um baixo risco para distúrbios respiratórios do sono e quanto aos hábitos de sono, 23 crianças (32,39%) apresentaram uma somatória menor, enquanto que 48 crianças (67,61%) ultrapassaram 41 pontos.

**Conclusão:**

Houve correlação entre risco para distúrbios respiratórios do sono em crianças com as queixas de alterações miofuncionais orofaciais e com a baixa qualidade/maus hábitos de sono.

## INTRODUÇÃO

Os distúrbios respiratórios do sono (DRS) são desordens que englobam aspectos caracterizados pela Síndrome de Resistência das Vias Aéreas Superiores (SRVAS), ronco, Apneia Obstrutiva e Central do Sono (AOS)^(1)^. Esta última se deve aos eventos rotineiros de bloqueio parcial ou total da passagem de ar no trato respiratório superior, em consequência de uma obstrução anatômica no tecido linfoide da faringe, favorecendo o seu colapso durante o sono^(2)^. Em crianças, as principais causas envolvidas se devem a fatores como hipertrofia de tonsilas faríngeas e palatinas, obesidade/sobrepeso, doenças neuromotoras e síndromes ou alterações do complexo craniofacial^(1-4)^. Tais aspectos podem comprometer o desenvolvimento global de crianças, principalmente em fase escolar, época de difícil estabelecimento de rotina de horários do sono, acarretando em prejuízos de crescimento ponderal e craniofacial, cardiopulmonares, neuropsicomotores, comportamentais, sonolência diurna excessiva, dentre outros transtornos e hábitos desfavoráveis ao sono reparador^(3,5,6)^.

O exame objetivo de referência mundial para estabelecer o diagnóstico de DRS é a polissonografia (PSG), entretanto, em decorrência de seu elevado custo e das dificuldades no acesso por grande parte da população mundial, a ciência nos últimos tempos vem avançando cada vez mais, para o desenvolvimento de questionários padronizados capazes de rastrear os riscos para DRS e também para outros transtornos do sono, o que parece favorável ao elencar as prioridades para realização do exame e a importância do tratamento precoce quando necessário^(7-11)^. Entretanto, os questionários de rastreio não substituem o diagnóstico realizado pela PSG.

Sabe-se que para os DRS obstrutivos pediátricos, o tratamento padrão-ouro é a adenotonsilectomia^(1)^. Todavia, os estudos mostram que mesmo após a realização da cirurgia muitas crianças continuam a apresentar alterações funcionais em seu sistema estomatognático, em decorrência do padrão orofacial antigo que possuíam antes da intervenção^(12)^, com alterações na estrutura craniofacial e na musculatura^(13)^, bem como nas funções de respiração, mastigação, deglutição e fala^(14,15)^.

Faz-se necessário portanto, uma avaliação miofuncional orofacial estruturada e um acompanhamento com realização de terapia miofuncional orofacial, mesmo após a cirurgia de adenotonsilectomia para que seja alcançada fundamentalmente, uma boa qualidade de vida para a população pediátrica^(12,15)^. Tendo em vista as questões acima levantadas, este estudo se propôs a correlacionar as queixas de alterações miofuncionais orofaciais e de risco para distúrbios respiratórios do sono com hábitos de sono na infância.

## MÉTODO

Trata-se de um estudo observacional, transversal, quantitativo no qual participaram 71 pais ou responsáveis de crianças de uma escola pública, do segundo ano pré-escolar ao quinto ano do Ensino Fundamental, no período de outubro de 2020 a abril de 2021. A faixa etária incluiu crianças de 6 a 11 anos de idade, de ambos os sexos. Os critérios de inclusão foram estudantes que estavam regularmente matriculados na instituição de ensino envolvida, possuíam idades entre 6 a 11 anos, às quais seus respectivos responsáveis tenham concordado com sua participação por meio da assinatura do Termo de Consentimento Livre e Esclarecido (TCLE) e o preenchimento de todo o formulário solicitado. A pesquisa foi aprovada pelo Comitê de Ética em Pesquisa em Seres Humanos da Universidade de Brasília - Faculdade de Ceilândia (UnB - FCE), sob o CAAE de número 33422820.4.0000.8093 e parecer de número 4.227.063. Foram excluídas do estudo, as crianças que utilizavam medicações para estimular o sono, possuíam diagnóstico de Transtorno de Déficit de Atenção e Hiperatividade (TDAH), Transtornos Invasivos do Desenvolvimento (TID) e síndromes que comprometessem o desenvolvimento craniofacial e/ou neurocognitivo. Além disso, aquelas cujos pais ou responsáveis não se voluntariaram, não assinaram o TCLE ou preencheram de forma incompleta os questionários solicitados também foram excluídas.

A coleta de dados foi realizada de forma remota, em uma única etapa, sendo aplicado um formulário por meio da plataforma *Google Forms.* A pesquisa foi adaptada para coleta de dados online devido às medidas de isolamento social para segurança no enfrentamento da pandemia de COVID-19. Desse modo, o contato inicial foi realizado via telefone com a coordenação da instituição de ensino participante, que realizou um levantamento dos escolares com a faixa etária estudada e se responsabilizou por encaminhar o formulário via *e-mail* aos pais/responsáveis das respectivas crianças. Sendo assim, todos os pais ou responsáveis das crianças matriculadas nesta instituição foram convidados a preencher questões semiestruturadas, contendo perguntas relacionadas à idade, sexo, peso, altura (calculando o Índice de Massa Corpórea - IMC), saúde geral, doenças do aparelho respiratório, medicamentos, aspectos alimentares, síndromes, dificuldades de mastigação e deglutição, fundamentalmente. No mesmo formulário, separados por categoria, também foram aplicados três protocolos, sendo eles: o *Nordic Orofacial Test-Screening* (NOT-S) - entrevista, traduzido e validado para o Português Brasileiro^(16)^, o *Pediatric Obstructive Sleep Apnea Screening Tool Questionnaire* (PosaST) - instrumento traduzido e validado para o Português Brasileiro^(11)^ e o *Children’s Sleep Habits Questionnaire* (CSHQ-PT) versão em português^(5)^.

O NOT-S é um protocolo que pode ser aplicado em diversas faixas etárias e funciona como um indicativo de queixas de alterações miofuncionais orofaciais. Possui duas partes, a saber: a entrevista inicial, que contém aspectos referentes à (I) função sensorial, (II) respiração, (III) hábitos, (IV) mastigação e deglutição, (V) salivação e (VI) secura da boca, e o exame clínico. Para a presente pesquisa, foi utilizada somente a entrevista, com o intuito de rastrear possíveis queixas oromiofuncionais. Dessa forma, cada domínio possui de um a cinco itens, que devem ser preenchidos com “X”, indicando presença de alteração, ou “0”, indicando ausência, podendo sofrer uma variação de zero a seis pontos (um a 12 para o protocolo completo), sendo as pontuações mais altas indicativas de alerta^(16)^.

O PosaST - versão brasileira possui alta sensibilidade para o rastreio de queixas de distúrbios respiratórios obstrutivos do sono em crianças em fase escolar. Consiste basicamente em uma entrevista/questionário para ser realizado com os pais/responsáveis pelas crianças com sintomas de distúrbios respiratórios do sono. Ele é composto por seis questões, cujo padrão de resposta é baseado na frequência com que ocorrem os episódios: “nunca” (0), “raramente” (1 vez por semana), “ocasionalmente” (2 vezes por semana), “frequentemente” (3 a 4 vezes por semana) e “quase sempre” (mais do que 4 vezes por semana). Entretanto, apenas no item sobre intensidade do ronco, a pontuação é fundamentada em: “baixo” (0), “um pouco alto” (1), “alto” (2), “muito alto” (3) e “extremamente alto” (4). Após isso, deve ser calculada a média das 6 questões, utilizando a fórmula: A = (Q1+Q2)/2; B = (A+Q3)/2; C = (B+Q4)/2; D = (C+Q5)/2. Para análise final deve ser considerado o escore somatório = (D+Q6)/2, Sendo Q1 referente à questão 1 e assim sucessivamente, sendo que a pontuação ≥ 2,72 representa um valor sugestivo de elevado risco para DRS^(11)^.

O CSHQ-PT é um questionário que foi adaptado para a Língua Portuguesa e possui a função de avaliar os hábitos do sono em crianças de 2 a 10 anos, por meio da percepção dos pais durante a semana anterior ou a mais próxima da avaliação. Essa versão é composta por 33 itens, que possuem 8 subescalas refletindo os seguintes domínios do sono: (1) resistência em ir para a cama (itens 1, 3, 4, 5, 6 e 8); (2) início do sono; (3) duração do sono (itens 9, 10 e 11); (4) ansiedade do sono (itens 5, 7, 8 e 21); (5) despertares noturnos (itens 16, 24 e 25); (6) parassonias (itens 12, 13, 14, 15, 17, 22 e 23); (7) distúrbios respiratórios do sono (itens 18, 19 e 20) e (8) sonolência diurna (itens 26, 27, 28, 29, 30, 31, 32 e 33). A frequência em que ocorrem os comportamentos é classificada em: habitualmente (5-7 vezes por semana, valor total 3 pontos); às vezes (2-4 vezes por semana, valor total 2 pontos) e raramente (0-1 vez por semana, valor total 1 ponto). Os escores dos itens 1, 2, 3, 10, 11 e 26 são inversos, sendo que a pontuação mais alta representa uma maior alteração do sono, e assim foi estabelecido que 41 pontos de corte seja capaz de detectar a presença de um distúrbio do sono^(5)^. Na análise, foi considerada a média e desvio padrão para a somatória de cada subescala, bem como a média do escore final.

Para a análise dos dados, a análise estatística descritiva foi expressa no formato de média, desvio padrão e respectiva porcentagem. Com o objetivo de correlacionar os achados das queixas de alterações miofuncionais, sono e distúrbios respiratórios do sono, foi aplicada a Correlação de Spearman entre os escores finais dos respectivos questionários NOT-S, CSHQ-PT e PosaST. O software utilizado foi o SPSS, versão 23 e o nível de significância adotado foi de 5%.

## RESULTADOS

Conforme a estatística realizada, foram incluídos 71 pais ou responsáveis de crianças. Dentre elas, 29 eram do sexo feminino (40,8%) e 42 do sexo masculino (59,2%), com média de idade de 8,52 anos. Em relação ao IMC, houve a média de 17,73±4,39 kg/m^2^.

Na anamnese houve relatos de problemas de saúde, apresentando maior ocorrência o relato de respiração oral noturna (15,5%), ronco (11,2%), asma (9,9%) e sinusite (8,5%).

Em relação ao protocolo NOT-S, as principais queixas miofuncionais orofaciais encontradas foram em relação aos domínios de respiração (35,2%), hábitos (33,8%), mastigação e deglutição (32,4%). Os resultados estão expressos na Tabela 1.

**Tabela 1:** Distribuição da amostra quanto às queixas miofuncionais orofaciais referidas pelos pais ou responsáveis a partir do protocolo *Nordic Orofacial Test- Screening* (NOT-S)

**NOT-S**	**Presença de queixa**
**Domínios**	
Função sensorial	13 (18,3%)
Respiração	25 (35,2%)
Hábitos (deletérios)	24 (33,8%)
Mastigação e deglutição	23 (32,4%)
Salivação	1 (1,4%)
Secura de boca	10 (14,1%)
**Total domínios**	**Média±dp**
	1,35±1,23

**Legenda:** NOT-S = *Nordic Orofacial Test -Screening*; dp = desvio padrão.

Foi possível verificar que todas as crianças apresentaram um baixo risco para DRS (Tabela 2) por meio da aplicação do PosaST. Enquanto que o questionário sobre os hábitos de sono CSHQ-PT expressou que, em média, esta população ultrapassou a nota de corte, caracterizando a possibilidade de distúrbio do sono (Tabela 3). Especificamente, 23 crianças (32,39%) apresentaram uma somatória menor, enquanto que 48 crianças (67,61%) ultrapassaram 41 pontos.

**Tabela 2:** Análise das questões do questionário *Pediatric Obstructive Sleep Apnea Screening Tool* (PosaST) quanto ao risco para distúrbios respiratórios do sono na infância

**Questões do PosaST**	**Média±dp**	**Mínimo**	**Máximo**
**Q1**	0,13±0,41	0	2
**Q2**	0,27±0,53	0	2
**Q3**	0,03±0,17	0	1
**Q4**	0,77±1,14	0	4
**Q5**	0,44±0,79	0	3
**Q6**	0,20±0,43	0	2
**Fórmulas preconizadas pelo questionário**			
**A**	0,14±0,40	0	2,00
**B**	0,04±0,19	0	1,25
**C**	0,32±0,69	0	2,62
**D**	0,38±0,67	0	1,75
**Escore total**	0,27±0,53	0	1,38

**Legenda**: dp = desvio padrão; Q1 = O(a) seu filho(a) para de respirar durante o sono?; Q2 = O seu filho(a) tem dificuldade para respirar durante o sono?; Q3 = Você alguma vez teve que sacudir seu filho(a) para fazê-lo voltar a respirar durante o sono?; Q4 = Com que frequência o seu filho(a) ronca?; Q5 = Você tem alguma preocupação sobre a respiração do seu filho(a) enquanto ele(a) dorme?; Q6 = Quanto de alto é o ronco do seu filho(a)?; A = (Q1+Q2)/2; B = (A+Q3)/2; C = (B+Q4)/2; D = (C+Q5)/2; Escore total = (D+Q6)/2.

**Tabela 3:** Distribuição da amostra quanto aos hábitos relacionados ao sono pelo questionário Children 's *Sleep Habits Questionnaire* (CSHQ-PT)

**Subescalas do CSHQ-PT**	**Média±dp**	**Mínimo**	**Máximo**
**Resistência em ir para a cama**	8,97±3,24	6	17
**Início do sono**	1,97±0,89	1	3
**Duração do sono**	3,83±1,37	3	9
**Ansiedade do sono**	6,07±1,89	4	12
**Despertares noturnos**	3,83±1,18	3	9
**Parassonias**	8,94±1,80	7	13
**Distúrbios respiratórios do sono**	3,62±1,06	3	7
**Sonolência diurna**	10,82±2,42	8	16
**Escore total**	44,90±8,20	33	66

Subescala (1) resistência em ir para a cama (somatória dos itens 1, 3, 4, 5, 6 e 8); Subescala (2) início do sono; subescala (3) duração do sono (itens 9, 10 e 11); Subescala (4) ansiedade do sono (itens 5, 7, 8 e 21); subescala (5) despertares noturnos (itens 16, 24 e 25); Subescala (6) parassonias (itens 12, 13, 14, 15, 17, 22 e 23); Subescala (7) distúrbios respiratórios do sono (itens 18, 19 e 20) e Subescala (8) sonolência diurna (somatória dos itens 26, 27, 28, 29, 30, 31, 32 e 33).

**Legenda:** dp = desvio padrão

A partir da análise inferencial, foi possível observar que houve relação positiva entre o risco de desenvolver distúrbios respiratórios obstrutivos do sono (PosaST) e a presença de queixas miofuncionais orofaciais detectadas através da entrevista contida no protocolo NOT-S (p=0,033). Além disso, verificou-se que a presença de risco de distúrbio respiratório obstrutivo do sono (PosaST) está relacionada com a presença de alterações no sono (CSHQ-PT) (p=0,007). Este por sua vez também se relacionou de maneira positiva com a presença de queixas miofuncionais orofaciais (NOT-S) (p=0,000). Os resultados estão descritos na Tabela 4.

**Tabela 4:** Correlação entre os escores dos protocolos *Nordic Orofacial Test- Screening* (NOT-S), *Pediatric Obstructive Sleep Apnea Screening Tool* (PosaST) e *Children’s Sleep Habits Questionnaire* (CSHQ-PT)

	**Média±DP**	**PosaST**	**NOT-S**	**CSHQ-PT**
**1.PosaST**	0,197±0,434		0,254	0,319
			0,033*	0,007*
**2.NOT-S**	1,535±1,519			0,438
				0,000*
**3.CSHQ-PT**	1,360±0,248			

Teste estatístico: Correlação de Spearman.

* Significativo = p<0,05

**Legenda:** PosaST = *Pediatric Obstructive Sleep Apnea Screening Tool*; NOT-S = *Nordic Orofacial Test Screening*; CSHQ-PT = *Children Sleep Habits Questionnaire* - Versão em português**;** dp = desvio padrão; primeiro dado foi o R = Coeficiente de proporcionalidade da correlação expressa, e na segunda linha o valor de *p* da Correlação de Spearman, considerando p<0,05.

## DISCUSSÃO

O presente estudo procurou levantar as queixas miofuncionais orofaciais relacionadas aos DRS de escolares, demonstrando correlação com as queixas de distúrbios respiratórios do sono e também com seus respectivos hábitos de sono. Ressalta-se então a relevância da atuação interdisciplinar da Fonoaudiologia com áreas correlatas de especialidade em sono, para diagnosticar e acompanhar crianças com queixas do sono, que podem coexistir com queixas das funções orofaciais.

De acordo com a literatura, crianças em faixa etária pré-escolar e escolar que possuem um risco para DRS costumam apresentar ronco^(3,17-19)^. Apesar da presente amostra ter sido de baixo risco para DRS, foi possível encontrar resultados similares aos da literatura.

Em relação às principais queixas miofuncionais orofaciais encontradas na Tabela 1, um estudo foi realizado com crianças brasileiras de oito a dez anos de uma escola pública, utilizando o NOT-S, porém sem associação direta com DRS. Verificou-se que na entrevista 50,8% das crianças apresentavam dificuldades para mastigar alimentos sólidos e o ronco estava presente em 24,4%^(20)^. Além disso, o estudo também mostrou que os domínios mais comprometidos foram mastigação e deglutição (50,5%), hábitos (41,4%) e respiração (26,4%)^(21)^, evidenciando que tais aspectos podem comprometer o desenvolvimento infantil. Destaca-se que apesar da não associação de maneira direta com os DRS, estes achados foram similares ao presente estudo.

Um outro estudo prévio foi realizado com crianças de 6 a 11 anos com diagnóstico de AOS e ronco primário, avaliando com o Protocolo de Avaliação Miofuncional Orofacial com Escores (AMIOFE), PSG e eletromiografia, e foi verificado que as crianças com AOS apresentam alterações de força/contração, postura, mobilidade de músculos mastigatórios e coordenação, o que desfavorece o bom desempenho das funções de mastigação, deglutição e respiração, sendo esta predominantemente oral^(12)^. Ressalta-se que no presente estudo não foram utilizados exames objetivos e protocolos de avaliação a fim de determinar o aspecto das funções orofaciais alteradas, e não houve divisão dos participantes em grupo experimental e grupo controle.

Com a aplicação da versão brasileira do questionário PosaST no presente estudo, verificou-se que todas as crianças apresentaram um baixo risco para DRS, conforme descrito no estudo de validação brasileira^(11)^. O questionário PosaST apresentou-se ser bastante sensível para rastrear principalmente os sintomas de DRS leve, que em 80% dos casos tende a melhorar com o uso de medicações e/ou terapia miofuncional orofacial, sem haver necessidade da cirurgia de adenotonsilectomia. A escolha deste instrumento para o presente estudo se deve ao fato de possuir alta sensibilidade e especificidade, ser um instrumento de rastreio e de fácil aplicação. No Brasil, até o momento não foram encontrados outros estudos de aplicação do PosaST - com exceção da validação brasileira - e como instrumento de triagem dos DRS foi encontrado somente um único questionário de rastreio, mas que além de incluir população escolar e pré-escolar, também inclui a população adolescente^(22)^, a qual foi excluída do presente estudo.

Os questionários relacionados aos hábitos e distúrbios do sono infantil foram elaborados como uma alternativa de triagem ao acesso à PSG e principalmente para complementar os achados da anamnese e avaliação clínica^(5,8,11)^. Dessa forma, foi considerado no presente estudo o termo DRS, pois segundo os *guidelines* internacionais e revisões sistemáticas é o termo utilizado para expressar um grupo manifestações respiratórias durante o sono capazes de provocar interrupção do fluxo ventilatório nas vias aéreas superiores, ocasionando microdespertares^(1,9)^. Especificamente na presente pesquisa, foi utilizado o questionário CSHQ-PT que sinalizou uma ocorrência de sinais indicativos de DRS menor do que a literatura traz^(3)^.

Foi observado também neste estudo que todos os protocolos utilizados no presente estudo apresentaram correlações entre si. Entretanto, não foram encontrados na literatura estudos que aplicaram pelo menos dois dos três protocolos juntos, sendo o presente estudo inovador neste quesito. Desse modo, foi possível observar que quanto maior o risco para presença de DRS, maiores serão os comprometimentos nas funções orofaciais. Apesar de não ter sido aplicado o PosaST e o NOT-S, um estudo realizado com 86 crianças brasileiras e italianas, na faixa etária de 4 a 11 anos de idade, utilizando outros instrumentos para rastreio de DRS e queixas orofaciais, verificou que crianças DRS apresentavam alterações no complexo craniofacial e consequentemente nas funções orofaciais, mostrando a relação existente entre os dois aspectos^(23)^. Além disso, outro estudo também evidenciou que há uma associação de queixas de DRS com alterações miofuncionais orofaciais^(24)^, como destacou o presente estudo.

Em relação aos aspectos abordados na relação expressa entre o PosaST e o CSHQ-PT, evidenciou-se que quanto maior o risco de DRS maior será o prejuízo ocasionado na qualidade de sono, o que se deve ao comprometimento nos padrões de latência, duração e eficiência do sono, em crianças com DRS, provocando sonolência diurna excessiva e múltiplos despertares, conforme os relatos da literatura^(1,6)^.

Na correlação expressa entre os escores do NOT-S e do CSHQ-PT foi evidenciado que a presença de perturbações no sono influencia de maneira direta no equilíbrio das funções orofaciais. Achados similares foram encontrados na literatura, como em um estudo brasileiro que utilizou o NOT-S para investigar as queixas miofuncionais orofaciais, todavia ao invés do CSHQ-PT foi utilizado o Índice de qualidade de Sono de Pittsburgh (IQSP); e observou-se que 38% da amostra apresentou uma baixa qualidade de sono^(25)^. Assim, todos os aspectos relatados anteriormente estão estreitamente interligados e contribuem para provocar um impacto negativo na qualidade de vida e no desenvolvimento de crianças em fase escolar^(5,26)^.

As limitações encontradas no presente estudo, deve-se principalmente ao fato de não conseguir realizar uma divisão das crianças em grupo controle e grupo experimental, tendo em vista que todas as crianças apresentaram um baixo risco para presença de DRS. Outro fator de grande impacto foi a impossibilidade de realização da coleta de dados de modo presencial - em decorrência das medidas de enfrentamento à pandemia de COVID-19 - com o intuito de utilizar a avaliação clínica contida no protocolo NOT-S, a fim de estabelecer-se com maior precisão a natureza das alterações orofaciais.

Para estudos futuros, ressalta-se a importância da utilização de avaliações e exames objetivos, a fim de determinar o diagnóstico das disfunções orofaciais em populações de maior amostra, bem como a realização da divisão entre grupo experimental e grupo controle, com o objetivo de melhor determinar a prevalência do risco para DRS e suas implicações na qualidade de sono infantil e características relacionadas ao sistema estomatognático.

## CONCLUSÃO

Houve correlação entre risco para distúrbios respiratórios do sono em crianças com as queixas de alterações miofuncionais orofaciais e com a baixa qualidade/maus hábitos de sono. As principais queixas miofuncionais orofaciais encontradas foram relacionadas ao sistema respiratório, visto que essas queixas podem estar associadas a um possível risco para presença de distúrbios respiratórios do sono, comprometendo o equilíbrio das funções orofaciais e da qualidade de sono de crianças em faixa etária escolar.
